# An Online, Moderated Peer-to-Peer Support Bulletin Board for Depression: User-Perceived Advantages and Disadvantages

**DOI:** 10.2196/mental.4266

**Published:** 2015-04-24

**Authors:** Kathleen Margaret Griffiths, Julia Reynolds, Sara Vassallo

**Affiliations:** ^1^ National Institute for Mental Health Research (NIMHR) Research School of Population Health Australian National University Canberra Australia; ^2^ National Institute for Mental Health Research (NIMHR, former affiliation) Research School of Population Health The Australian National University Canberra Australia

**Keywords:** Internet, support group, mental health, depression

## Abstract

**Background:**

Online, peer-to-peer support groups for depression are common on the World Wide Web and there is some evidence of their effectiveness. However, little is known about the mechanisms by which Internet support groups (ISGs) might work.

**Objective:**

This study aimed to investigate consumer perceptions of the benefits and disadvantages of online peer-to-peer support by undertaking a content analysis of the spontaneous posts on BlueBoard, a well-established, moderated, online depression bulletin board.

**Methods:**

The research set comprised all posts on the board (n=3645) for each of 3 months selected at 4 monthly intervals over 2011. The data were analyzed using content analysis and multiple coders.

**Results:**

A total of 586 relevant posts were identified, 453 (77.3%) reporting advantages and 133 (22.7%) reporting disadvantages. Positive personal change (335/453, 74.0%) and valued social interactions and support (296/453, 65.3%) emerged as perceived advantages. Other identified benefits were valued opportunities to disclose/express feelings or views (29/453, 6.4%) and advantages of the BlueBoard environment (45/453, 9.9%). Disadvantages were negative personal change (50/133, 37.6%), perceived disadvantages of board rules/moderation (42/133, 31.6%), unhelpful social interactions/contact with other members (40/133, 30.1%), and technical obstacles to using the board (14/133, 10.5%).

**Conclusions:**

Consumers value the opportunity to participate in an online mutual support group for mental health concerns. Further research is required to better understand how and if these perceived advantages translate into positive outcomes for consumers, and whether the perceived disadvantages of such boards can be addressed without compromising the safety and positive outcomes of the board.

## Introduction

Peer-to-peer Internet support groups (ISGs) are an accessible source of support and advice for health conditions. According to the Pew Internet study, 18% of Internet users have searched online for others “with health concerns similar to theirs” [1]. ISGs which enable users to communicate with their peers anonymously may be particularly attractive to those with stigmatized conditions such as depression [2], particularly where the condition is treatment resistant.

A recent randomized controlled trial found that a depression ISG was effective relative to an attention control group in reducing clinically significant depressive symptoms over a 6-month period [3]. Little is known about the mechanisms by which such an improvement might occur. However, some insight into these processes might be provided by a consideration of consumer perceptions of the benefits and disadvantages of Internet support groups.

Several studies have provided data on consumer-perceived benefits of depression ISGs using data from quantitative surveys [4-7]. Reported advantages from these survey studies included emotional support [5,6], an outlet for expression [5], the opportunity to talk about matters that could otherwise not be discussed [4], reduced isolation [4,5], and information about medication [4]. Other documented advantages included improved symptoms [6] and increased formal help seeking [4]. In a study which undertook a factor analysis of items measuring perceived advantages of ISGs, Nimrod and colleagues [7] reported two factors: offline improvements, in the form of improved daily functioning, and online advantages, in the form of social support. However, several items cross-loaded on both factors.

A limitation of the studies used by authors to date to investigate perceived advantages of ISGs is that they have all relied on the subset of ISG users who are prepared to respond to a survey. In addition, such surveys are devised by researchers rather than based on users’ reports of benefits. It is, therefore, possible that they do not encompass all user-perceived advantages of ISGs. An alternative approach to investigating the benefits or problems with ISGs is to analyze statements of benefit and disadvantage in spontaneous posts (ie, user messages) on a support forum. To our knowledge, only one published study has reported the results of such an investigation. Horgan, McCarthy, and Sweeney [8] investigated the posts of university students who posted anonymously on a researcher-moderated depression ISG. Reported benefits based on a qualitative analysis included “sharing their feelings,” “a sense of not being alone,” a “shared understanding,” and “anonymity.” However, the analysis was based on only 56 posts by 13 participants on an experimental ISG. Further, the support group was generated for the purpose of the research and was available for only two university terms. Thus, it is unclear if the findings would be applicable to a more heterogeneous target group with respect to age and background, and to a more established, open, and publicly available support group. In addition, the study did not explicitly investigate the potential disadvantages of the support group.

This study aimed to investigate the perceived advantages and disadvantages of an online ISG by analyzing posts sampled over several periods of time from a well-established, moderately large, publicly available mental health ISG [9]. This ISG was open to any person aged 18 years or older, regardless of demographic background or country of residence.

## Methods

### Overview

**Figure 1:**
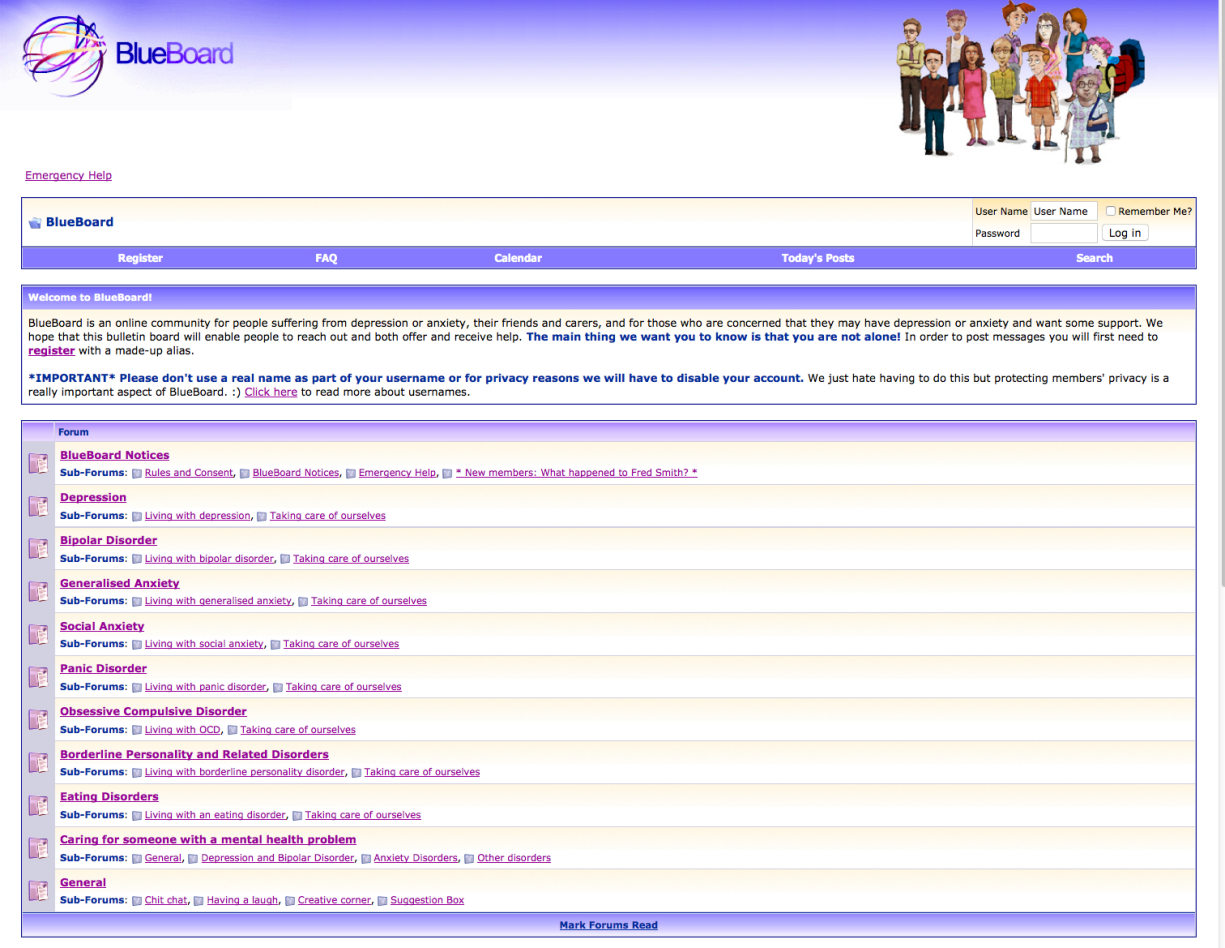
BlueBoard homepage.

BlueBoard is a moderated, online peer-to-peer support group for mental health problems, including depression, bipolar disorder, anxiety, and borderline personality disorders [9] (see Figure 1). The majority of the posts are made on the depression forum. BlueBoard is run as a service by the National Institute for Mental Health Research with funding from the Australian Department of Health. BlueBoard moderators—known as the Mod Squad—are trained consumers overseen by an experienced registered clinical psychologist (JR). The moderators are tasked with ensuring that users interact in a respectful manner and adhere to the rules of BlueBoard. The rules do not allow members to post potentially identifying details or explicit references to suicide, self-injury, and harm to others. The moderators monitor posts and, where necessary, remove material that does not comply with the rules. The moderators do not, however, participate as members. Although BlueBoard is a formal service rather than a research intervention, all members of BlueBoard consent to the analysis of BlueBoard posts at the time of joining the board, according to an Australian National University (ANU) Human Research Ethics approved protocol. Members learn of the board primarily through online searches and links from other websites.

The data were extracted in the form of board posts and reported posts, the latter being messages sent to the board moderators, for example, when reporting a post the member perceived to be inappropriate. These data were analyzed using content analysis, multiple coders, and an inductive approach [10].

### Coding

#### Overview

The coding categories for positive and negative experiences on BlueBoard were developed using a multiple-step approach commencing with a set of data not used in the final coding.

#### Training Sets

In the first instance, three researchers (JR, SV, LB) independently identified and developed a classification system for posts in Training Set 1, which consisted of all posts for October 2010 (n=1220). The coders subsequently discussed and agreed on a common classification system for the positive and negative posts. Two of the coders (JR, LB) then recoded the posts according to the common classification system, further refining it by consensus.

Training Set 2 consisted of all posts for September 2010 (n=1295). The refined system was then tested by the two raters on approximately half of the posts in Training Set 2 (599/1295, 46.25%). Since the raters found it difficult to code the large dataset directly into categories, a two-stage coding process was developed and tested on the second half of the posts in Training Set 2 (696/1295, 53.75%). In phase 1, posts were first scored as relevant or not relevant. Discrepancies were resolved by consensus. In phase 2, the posts were allocated into categories and subcategories with final coding by consensus.

#### Research Set

The research set comprised all posts (n=3645) for each of 3 months selected at 4 monthly intervals over 2011. Posts not relevant to the study, such as duplicate posts and material posted by moderators and spammers, were removed (n=1928). This left 1717 posts—April (n=598), August (n=509), and December (n=610). These posts were rated for relevance by two raters (JR, SV) with discrepancies resolved by consensus. The latter were imported into NVivo 10 and rated according to the categories and subcategories developed in the training sets. The resulting coding scheme was subsequently refined at the category and subcategory level by the first author (KG) using NVivo 10. For the purposes of reporting, each post was rated according to whether it contained content relevant to each of the categories. The final coding was checked by a second rater (JR) with the few discrepancies (n=5) resolved by consensus. Quantitative data (ie, frequencies) are reported for the top-level categories.

## Results

### Overview

A total of 586 posts were found by rater consensus to refer to the advantages and/or disadvantages of participating in BlueBoard. A total of 212 members contributed to the total research set and 103 to the posts rated as relevant. Of the latter, 97 provided demographic data at registration of whom the majority were women (74/97, 76%), lived in Australia (89/97, 92%), and resided in a city (77/97, 79%). Participant age was recorded in age bands. Using midpoints of the bands for the purpose of calculation, mean participant age was found to be 37.2 (range 18-19 years to 60-65 years). Of the 94 participants who provided information about their clinical status, 69 (73%) were consumers.

### Findings

Of the 586 relevant posts, the majority (453, 77.3%) reported advantages of the board. The remainder were concerned with disadvantages. The themes extracted for each are reported in Table 1, are summarized in turn below, and are illustrated by quotations derived from user posts. Spelling errors in these quotations have been edited for ease of reading.

**Table 1:** Broad themes emerging from the content analysis of BlueBoard: advantages and disadvantages.

Broad themes		Number of posts, n (%)
**Advantages (n=453)**		
	Positive personal change	335 (74.0)
	Valued social interactions and support	296 (65.3)
	Valued opportunities to disclose/express feelings or views	29 (6.4)
	Other advantages of the BlueBoard environment	45 (9.9)
**Disadvantages (n=133)**		
	Negative personal change	50 (37.6)
	Perceived disadvantages of board rules/moderation	42 (31.6)
	Unhelpful social interactions/contact with other members	40 (30.1)
	Technical and perceived obstacles in using the board	14 (10.5)

### Advantages

As noted in Table 1, four broad themes emerged from the posts about the advantages of the support group, including positive personal change, valued social interactions and support, valued opportunities to disclose/express feelings or views, and other advantages of the BlueBoard environment. Since many posts contained multiple elements and were, therefore, coded into more than one broad theme, the values in Table 1 do not sum to 100%.

#### Advantage 1: Positive Personal Change

##### Overview

One of the top two themes to emerge from members’ posts related to positive personal changes associated with participating in the board. Many members reported nonspecific positive changes—“I post a lot on this site and it helps that’s why I do it” [Participant #1]. In addition, specific emotional, cognitive, and behavioral changes were reported as illustrated in the following sections.

##### Emotional Effects

Members reported positive emotional changes in the course of using the board. Sometimes these referred to a nonspecific effect of the board—“Thanks so much, believe it or not that make me feel a lot better” [Participant #2]. However, often the emotional change reported was specific and involved a current, rather than long-term, effect.

The most common specific emotional effect was gratitude or appreciation with the posts incorporating either general—“Thanks heaps” [Participant #3]—or targeted expressions of appreciation—“Thanks guys, appreciate your kind words…” [Participant #4].

Members also expressed happiness or feeling glad:

Thank you for your support and caring words...brought a smile to my face :).

Participant #1

Glad to hear you’re feeling better.

Participant #5

Members also derived amusement from posts on the board:

LOL, very funny! Thanks for sharing that :).

Participant #6

hahahhah hilarious!

Participant #7

*rofl.* (rolling on the floor laughing) [Participant #8]

Some participants expressed a sense of relief—“Phew, it’s normal” [Participant #9]. Typically members did not discuss the effect of the board on their mood specifically, although one member noted, “I don't know if it’s related, but my mood has actually been better these past few days I've been on here!” [Participant #10].

Members also reported experiencing hope as a result of hearing the stories of others:

I love to hear about others healing and journey, helps me keep on track and gives me hope. :)

Participant #7

Ever seen the movie Pay It Forward? Hope given, and hope returned... I think that's what this place is all about.

Participant #11

It is good to know that there is some sort of light at the end of the tunnel.

Participant #12

Further, the board was seen as a source of inspiration:

I want to thank you as well, I felt very inspired by your story.

Participant #13

Maybe we need our own little motto in here... inspired by you...

Participant #12

WOW!!!!! That is inspirational, I'm in the right head space to be able to truly believe that.

Participant #9

##### Cognitive Effects

Use of the board was also associated with self-reported change in members’ thinking.

Posts on the board were seen as enhancing the member’s knowledge—“Have read this forum since I was diagnosed in Jan and have found the information very informative” [Participant #14]. Through exposure to the board, members also developed the knowledge that they were not alone—“It's nice to know that I'm not alone, and that other people are going through the same thing” [Participant #10]. Further, posts were perceived as thought provoking—“Your post really made me think” [Participant #15]—and as providing insight and a different perspective:

Both of you have [made] me realize opening up to him is a good idea and is not weak :).

Participant #1

I did not think of it in this way but maybe you are right.

Participant #16

Supporting others was seen by some members as a way of helping themselves by thinking through their own circumstance or reminding and motivating themselves to maintain their aims:

I really thank you for your post on this forum as my post has been as much about thinking about why I'm feeling good, as it is providing advice to you.

Participant #17

Actually, helping others helps me, because it reinforces all of the things I have learned, keeps them fresh in my mind so I don't slip.

Participant #15

Finally, participants reported that the posts on the board stimulated the intention to act:

That’s a really good idea...thanks for your advice, I’m going to try that the next time I’m in the situation :).

Participant #18

Thanks for the advice I'll try writing a journal and see how I go.

Participant #19

##### Behavioral Effects

The use of BlueBoard was also associated with behavioral changes. This included evidence of acting on a suggestion on the board. In some cases, the suggestion involved a recommendation that the member consult an information source:

You recommended a book called "The Happiness Trap" by Dr Russ Harris. I’ve just started reading it and am finding it extremely helpful and informative, thank you so much for passing on your knowledge :).

Participant #7

In other cases, the behavioral effect involved a help-seeking act:

Thanks for your help guys. I went and saw a new doctor this morning with my mum’s support as I couldn't face seeing someone new on my own. He has prescribed me medication and swapped me to a different counsellor and has told me if I don’t connect with him he will change me to someone else. I guess I just needed that little push to make a change as I am not good with change.

Participant #30

#### Advantage 2: Valued Social Interactions and Support

##### Overview

I don't know why I didn't join one of these discussion boards earlier, the support from everyone is amazing!

Participant #10

The second major theme to emerge from the analysis was that BlueBoard provided an opportunity for its users to engage in valued social interactions and to receive support. Members frequently expressed gratitude to other BlueBoard members for their responsiveness—“Thank you again for replying, you have no idea how much it means to me that someone did because I was so scared” [Participant #20]. Other posts referred to the culture of the board as one of support or mutual support:

You need to understand that you will have support from everyone on here.

Participant #21

We are all here to support each other.

Participant #22

Although there were generic references to support—“A big thank you to everyone who posts on BlueBoard. Your thoughts and words are valuable and appreciated” [Participant #11]—many of the posts pointed to specific types of social support, such as shared understanding, a nonjudgmental environment, advice, information, and emotional support and companionship. Each is described separately below, but we acknowledge that these concepts are interlinked, rather than mutually exclusive.

##### Shared Understanding

A key perceived advantage of the board was that it provided its members with the opportunity to interact with others with a shared understanding of living with mental health problems*:*

It's like a breath of fresh air to find this BlueBoard and communicate with people who truly understand and relate to what I'm going through.

Participant #9

You can talk openly...and the members really get what you’re going through because they have been there themselves.

Participant #23

This shared understanding in turn was perceived as validating*,* reducing the sense of isolation, and enhancing a sense of belonging:

Depression makes you feel totally isolated and detached, and you convince yourself that no-one else could possibly understand how you feel. This site has been a blessing for me because it gives me a sense of “belonging”...I guess it makes me feel a little less like a freak, knowing there's a whole bunch of other "freaky" people out there, who are just as normal as everyone else, aside from our common illnesses. In a society that judges, and bullies, and stigmatises everything, this place is a welcome reprieve.

Participant #11

##### Nonjudgmental Environment

As illustrated in the latter and other posts, this shared understanding was also seen as fostering a nonjudgmental (ie, nonstigmatizing) and, therefore, emotionally safe space:

I'm so glad that you've found this forum, it's a great way to express how you feel without judgment and get support from others who know the place you’re in.

Participant #24

It’s a lovely safe haven where no one judges you.

Participant #11

##### Emotional and Companionship Support

In addition to the support arising from the validation and sense of belonging associated with a shared understanding of depression/mental health problems, the analysis of posts indicated that participants valued a number of other forms of emotional and/or companionship support on the board, including kindness, caring, comfort, warmth, understanding, encouragement, self-esteem support, and friendship (see Table 2).

**Table 2:** Examples of emotional and companionship support reported on BlueBoard.

Emotional and companionship support	Examples
Kindness	“You're another special presence on this board. A silent sufferer who has so many kind and loving words for others” [Participant #11].
Caring	“Just as [BlueBoard member] above said, we care what happens to you, even though it's virtual friendship doesn't mean we don't care because we do” [Participant #8].
Comfort	“Thanks for that - yes I did read (and take comfort) from the other post you mentioned” [Participant #25]. “oooo nice cuddle, thanks :)” [Participant #7].
Warmth	“I love that when there is a new member intro, [Member 1] and [Member 2] are usually the first to welcome them in the warmest way!” [Participant #7].
Understanding	“Welcome [new BlueBoard member], you'll find lots of support understanding and advice on offer here on BB. Good on you for joining, not always an easy thing to do, but you've done it” [Participant #8].
Encouragement	“You have no idea how much encouragement you've given me, as a sufferer of this stinking illness” [Participant #11].
Self-esteem support	“Dear [lists 7 BlueBoard members] and all who read this post, I thank you for all the compliments and or feedback” [Participant #26].
Friendship	“You will find virtual friendships of all kinds here, you sound like you have plenty of experience to draw from. You will find plenty of us with similar stories of isolating ourselves for all sorts of reasons” [Participant #8].

##### Advice, Wisdom, and Informational Support

A frequently cited specific form of social support was advice from other members:

That’s a really good idea...thanks for your advice, I’m going to try that the next time I’m in the situation :).

Participant #18

Your advice is always so good... :o.

Participant #27

A minority of these posts specifically concerned advice designed to promote help seeking from a professional—“Thanks for the tips about seeing a counsellor” [Participant #28]—from another service—“Thanks also for the idea of disability employment organisations and a case worker:)” [Participant #13]—related to the provision of information—“Thanks for letting us know of the program, I didn’t manage to catch the insight program but will see if I can access it online” [Participant #29]—or by sharing wisdom—“Thank you so much for the wisdom that you have shared, I really appreciate it.:)” [Participant #9].

#### Advantage 3: Valued Opportunities to Disclose/Express Feelings or Views

The board was seen as a safe place for members to express their feelings or views. In particular, a number of members referred to the importance of the board as a place to vent—“It's amazing how it helps to vent, just getting things said and off your chest can make the burden seem just that much lighter” [Participant #8]. This was not necessarily in expectation that others would respond or solve the problem:

I don't want words of sympathy or support or comfort. I just need to say this.

Participant #30

I’m not really expecting anyone to reply...I just really need to get some things out otherwise I feel like it will completely take over me.

Participant #19

Posting was seen as an alternative to ruminating on the problem—“I guess I'm just sick of not talking about it and being stuck inside my head” [Participant #17]—and as a place where members would not be judged for their words—“I personally have found this forum to be good to express how I feel at any given time [without] ridicule or judgment” [Participant #9]. The board also provided a means of venting safely without burdening those closest to them—“I need a place to vent so my poor husband doesn’t have to listen to my constant whining and misery I just need someone to hear me and not call me crazy!” [Participant #31].

#### Advantage 4: Other Advantages of the Board Environment

Other posts related to the advantages of the BlueBoard environment and its accessibility. The board was seen as providing a means for people to reach out—“This is a good site. I too never thought of reaching out on something like this but its been fantastic” [Participant #32]. Some members saw the board as an accessible alternative when face-to-face or therapist support was unavailable:

We are here for you so you can always count on us. I know that it is not the same as seeing someone face to face and particularly your psychologist but it is better than having no support at all.

Participant #16

I was supposed to be seeing my psychologist this afternoon but now I can't so I thought I'd talk to you instead lol :rolleyes:.

Participant #33

The psychologist forgot to turn up today, a day when I could really do with seeing him...hence why I am on here.

Participant #34

Some felt the board had advantages over face-to-face interactions, providing a safer and less threatening and constraining medium to communicate about their illness:

No forums aren’t the same as talking face-to-face, but sometimes it’s easier to just simply be yourself and open up without having to worry about being judged face to face.

Participant #35

I don't exactly tell people about this face-to-face but that is what this forum is for. And we certainly won’t judge you.

Participant #15

I am much more talkative and eloquent in here than in real life.

Participant #36

Safer to vent here than actually act on it in the real world.

Participant #15

The Internet support group was also seen by some as a preferable format to social media sites such as Facebook:

[A social network]...it’s not that comfortable and I find it difficult to write what I'm really thinking, feeling...

Participant #13

I’d rather listen to all your honest voices than the not so honest perceptions I often find on the dreaded Facebook.....lol.

Participant #37

One member who had been refused admission to another online website noted of the board, “So it’s nice to know there are sites out there that are willing to accept anybody and listen” [Participant #20].

Some members referred to the special qualities of other members—“It’s very nice to have places like this, where people are genuine, honest and understanding. I think having this condition is very leveling and makes superficial, ego boosting conversation tiresome, just doesn't feel good” [Participant #13].

The availability of the posts as a record was seen as an asset. For example, members noted that lessons could be learned from consulting both one’s own and others’ past posts:

Before you make any rash decisions about going off your meds...do yourself a favour and read through your old posts...just to remind yourself what happened last time.

Participant #11

All I can suggest is have a read through some of the posts on this site. It may give you a little bit more of an insight into what we go through on a daily basis and may help you to understand a little bit more about depression.

Participant #32

The board was seen by one member as a potential facilitator of communication with a health care professional—“Perhaps you could print out your posts and give them to your GP to read if you have trouble talking” [Participant #8].

Another advantage of the format of the board was that even when people were not well enough to participate fully on the board, they were able to follow the progress of others by monitoring the board—“Haven’t been too far away...always keeping an eye on things and following your progress...just wasn’t up to participating for a while” [Participant #11].

Finally, members expressed appreciation for the role of the board moderators, known as the Mod Squad:

Thanks for...the time and effort you put in to keep this board going, so that we have somewhere to come and share and support one another. Much appreciated.

Participant #11

I think the mods will continue to keep up the good work in keeping things, well, moderate here.

Participant #8

### Disadvantages

#### Overview

A minority of relevant posts (133/586, 22.7%) were concerned with the disadvantages of the board. Four themes emerged: negative personal change, perceived disadvantages of board rules/moderation, unhelpful social interactions/contact with other members, and technical and perceived obstacles in using the board (see Table 1).

#### Disadvantage 1: Negative Personal Change

There is potential for the descriptions of negative experiences to cause distress to those reading them. A number of members indicated that they were sorry, sad, or worried to learn of another member’s problems:

It’s really upsetting to hear how you are struggling with it all...as people on this site we know it’s hard.

Participant #1

I'm really worried about you.

Participant #38

It is likely that many of these posts were expressions of empathy and reassurance, rather than deep personal emotional distress—“I'm so sorry to hear things aren’t improving” [Participant #11]. On the other hand, one member was clearly distressed by content posted on the board, content that was against the BlueBoard rules. The member read the material before it was removed by moderators and experienced the post as “triggering” and asked the other member to “Stop writing about your overdoses!!!!!! I can’t handle it!!!!!!” [Participant #39].

Some members expressed concerns about how their posts might be received by, or have affected, others:

By the way I'm having a really crappy day today so I hope I don’t sound cross, I'm not.

Participant #15

I was worried I would be hammered by writing this :P.

Participant #40

Oh sorry, I obviously got confused! Sorry for any offense.

Participant #7

I worry a lot about being accepted (I’m even worried about posting this).

Participant #41

One member who used humor to cope with their depression wrote, “BTW, if anyone has a problem with the manner in which I project my depression, please do let me know, I do not wish to upset anyone!” [Participant #42].

Finally, some members expressed frustration and irritation with the BlueBoard rules—“So Mr/Mrs Moderator. I am very annoyed” [Participant #30] (see theme in the next section).

#### Disadvantage 2: Perceived Disadvantages of Board Rules/Moderation

For reasons of safety, BlueBoard is governed by strict rules and careful moderation according to preestablished protocols. This was seen as unnecessarily restrictive by some members—“It may take you a while to get used to the rules of this website. I have had substantial trouble given that as a creative person I tend to dislike rules and be a bit deviant at times” [Participant #16].

For example, some participants perceived the BlueBoard rule that members should not discuss self-harm or suicide as a limitation of the board—“I understand these sites have a certain ‘duty of care.’ Having said that, I read an article in the paper yesterday which made this comment: ‘It has been found that talking about suicide does not cause it to happen, and not talking about it does not prevent it’” [Participant #11]. Moreover, a strongly felt need to speak about suicide or suicidal thoughts led to attempts by some members to circumvent the rules by expressing their thoughts indirectly—“Dark thoughts which I know we’re not supposed to talk about” [Participant #43].

Other members were frustrated when sections of their posts that did not conform to the rules were edited by the moderators—“What’s the point of trying to express myself if you’re going to cut bits of my expression out?” [Participant #30]—or removed—“Kinda spewing. My other whole post got wiped out” [Participant #36]. Perceptions that participants were treated unequally were also an occasional source of complaint—“Why is this still here and my post deleted? It’s not fair” [Participant #39]. A small number of members knowingly broke the rules to make a post that they hoped would be read by other members before the moderators removed it, which resulted in placing the members’ accounts on delayed “telecast” (ie, premoderation).

To ensure members are not identifiable, BlueBoard requires registration with an alias that is not the name of any person since the moderators have no means to determine that a real name is not the name of the member. However, this rule confused some members:

You've disabled my account under the name [deleted]. I assure you this is a random name I saw in a book once and I like it. It isn't even close to my real name. Could you please reinstate it?

Participant #44

Other members were disappointed or annoyed that they were unable to easily contact the moderators, or alternatively felt that it was “Pointless trying to talk to a faceless moderator” [Participant #30].

Finally, some members felt the asynchronous nature of the board was a limitation. BlueBoard does not have a real-time chat facility due to the challenge and resources that would be required to moderate it. In addition, for the safety of the members, the board does not permit posting of links to other providers. However, some felt that BlueBoard should introduce a chat room or refer members to appropriate external providers:

There are very few safe places online for people like us to meet, and while BlueBoard brings us together, it’s frustrating not being able to converse with other members. Surely as adults we have the right to contact other like-minded folk, and can take responsibility for the outcome of those choices without holding BlueBoard to ransom. A disclaimer on the BlueBoard site would surely cover that? Or am I being naive?”

Participant #11

#### Disadvantage 3: Unhelpful Social Interactions/Contact With Other Members

Although all members on BlueBoard register anonymously, one member reported that they believed they were being stalked on the board by a person from their offline life with whom they were in conflict. This accounted for 26 posts (65%) of a total of 40 in this theme. Unsure if the alleged stalker was restricting themselves to reading, as opposed to contributing, BlueBoard posts, the member wrote, “I hate to think this but one of you who even post to me may be the stalker” [Participant #45]. The member concluded, “I will never post again, as I don't need [them] knowing about my life. I actually felt safe here” [Participant #45].

Other concerns, each of which was identified in 1 to 3 posts, were negative debate, unanswered posts, disputed information or advice, and misinterpreted communications. Finally, a small number of members felt that they did not “belong” on BlueBoard due to their current mental health status, age, or other attributes. For example, one member who had recovered was concerned about the impact their happiness might have on others on the board, and another member was unable to identify with others on the board—“I doubt anyone walks my footsteps. I've read through the first page of posts here and I seem to be the most violent person here” [Participant #30].

#### Disadvantage 4: Technical and Perceived Obstacles in Using the Board

A small number of posts referred to difficulties in using the board, including not knowing how to post to the board, losing a post whilst in the process of composing it because they exceeded the board’s automated time-out period, and prematurely sending a message before it was completed. Other perceived obstacles to board use each noted in 2 posts included difficulty in communicating emotional nuances online and difficulty in writing posts when depressed or anxious.

## Discussion

There were both perceived advantages and perceived disadvantages of the board environment. These are discussed in turn in the following sections.

### Advantages

Based on the posts by members, the board provided valued social interactions and support, including shared understanding, a nonjudgmental environment, advice, and informational, emotional, and companionship support. The latter included kindness, caring, comfort, warmth, understanding, encouragement, self-esteem support, and friendship. The board was also associated with positive personal change, including specific emotional, cognitive, and behavioral effects. Further, it enabled participants to express or vent their feelings. Other advantages of the board environment were that it provided a place for members to reach out, was an accessible alternative to face-to-face support, particularly when the latter was unavailable, was preferable to social media sites, and had members with special qualities. Further, the availability of the posts as a reference or for facilitating communication with the members’ doctors were seen as strengths of the board.

To our knowledge, these findings represent the first comprehensive evaluation of the perceived benefits of a publicly available, depression-related support group based on members’ posts. The results are consistent with, but considerably extend, the findings of the small pilot study of student posts on a closed experimental board reported by Horgan et al [8]. The latter reported some themes that were categorized in the current study as valued social interactions and support. However, the paper did not explicitly discuss other strengths, such as a nonjudgmental environment, advice, or informational, emotional, or companionship support. Nor did it identify personal change or most strengths of the board environment. It is possible that this reflects the limited duration and number of posts on the student board and, hence, a lack of a strong board culture or trusted social network. Horgan et al [8] did, however, report that anonymity was a valued attribute of the student board. This factor did not emerge in this study. It is possible that such anonymity is more highly valued by younger than older people, or by members who share a physical environment where they may be known by, or physically encounter, other members.

The advantages of the board documented in this study point to possible mechanisms for observed improvement in depression outcomes associated with an Internet support group [3]. For example, improvements in depression might be mediated by emotional support or advice, or information provided by participants which facilitate coping or help seeking. This is consistent with findings from an unpublished quality assurance survey of BlueBoard members, half of whom indicated that they were more likely to access other forms of help, such as consulting a doctor or therapist, as a result of using BlueBoard.

Our own previous research has documented an increase in perceived emotional and informational social support following the use of an online support group [11]. However, it also found an increase in perceived social support of similar magnitude among participants in the control condition [11]. It is possible that although members value social support, it does not mediate improved depression outcomes. Alternatively, the scale may have failed to measure elements of online social support that might be critical to improved mood. For example, the scale did not explicitly measure shared understanding or access to nonjudgmental support. Future outcome research on ISGs should employ items and scales that measure attributes which users consider to be positive aspects of the board and investigate their role in mediating change in mental health outcomes.

Many of the elements identified in this study are consistent with the therapeutic factors outlined by Yalom and Leszcz [12] to explain the positive effects of group psychotherapy. These include the installation of hope, universality, imparting information, altruism, the development of social skills, interpersonal learning, group cohesiveness, and catharsis. ISGs such as BlueBoard may also be conceptualized as supporting processes critical to broader mental health recovery. For example, a recent systematic review identified five key processes in recovery: connectedness, hope/optimism, identity, meaning, and empowerment [13]. The advantages of participation spontaneously expressed by BlueBoard members are highly consistent with these processes, particularly connectedness, hope/optimism, and identity. Future research should incorporate measures relevant to Yalom and Leszcz’s [12] therapeutic factors and to recovery outcomes in addition to measures of specific symptoms such as mood.

### Disadvantages

Members also identified some potential disadvantages associated with the board. This included negative emotional changes associated with concern for another member of the board, a member’s concern about the possible impact of their posts on others, and frustration at the board rules. Some members perceived disadvantages of the board environment, including the restriction imposed by the board rules and the effects of moderation (eg, that posts on the topic of self-harm and suicide were not permitted, nonconforming posts were edited, participants were required to register using an alias that was not a name, and that the format of the board did not include a synchronous service). A small number technical obstacles were also encountered by members using the board. Finally, there were some instances of unhelpful social interactions/contact with other members, including a report of alleged stalking on the board. Other unhelpful interactions did not account for a large number of posts.

To our knowledge, this is the first systematic analysis of the disadvantages of a publicly available, depression-related support group based on members’ posts. The advantages substantially outweighed the disadvantages of the board. However, by definition the posts on the board would have been weighted toward the perspectives of members who remained active on the board and, thus, might overestimate the advantages relative to the disadvantages of the ISG. Regardless of the magnitude of the problem, the identified problems do raise important questions.

The board enforces strong rules with a view to ensuring the safety of its members. The fact that very few members complained of unsafe or negative interactions with others on the board may reflect the benefits of such rules. However, little is known empirically about the impact of such rules on members or their mental health. Research to investigate such questions would raise significant ethical considerations. For example, although it would be of academic and practical interest to compare the effects of a moderated board with those of an unmoderated board, the ethics of undertaking a trial, were it to entail randomization, would require careful consideration. A study might be undertaken with the cooperation of the owner of an unmoderated board to compare the effects of a moderated and an unmoderated board. However, the meaningfulness of such a comparison would be limited by the methodology employed.

As a first step, there may be value in analyzing the posts on an unmoderated board of a similar size to investigate the perceived advantages and disadvantages of this type of board, and to compare them with those documented here. Unfortunately, it is unlikely that registrants on an unmoderated board will have provided prior ethics releases for research on their posts and it would not be feasible to obtain retrospective permissions for all past participants. Accordingly, such research raises moral and ethical issues even in a publicly available board. If these concerns can be addressed, there may be considerable benefits to undertaking such a comparative study.

### Limitations

For pragmatic reasons, this study analyzed only a small subset of the messages posted on the Internet support group over a relatively short time frame. The primary themes emerged strongly, suggesting that the sample was adequate for the time period targeted. However, themes or their relative importance may change over time as a support group matures. Therefore, this data may not be applicable to all the developmental phases of an online support group. Further, as noted above, the posts of those who have left the board—both those who were dissatisfied with the board and those who have recovered—may be underrepresented in the data. Finally, the analysis focused on one ISG only, with a preponderance of members from one country—it is unclear if the findings will generalize to other online depression support groups comprising citizens from other countries or cultures.

### Conclusions

Consumers value the opportunity to participate in an online mutual support group for depression. Further research is required to better understand how and if these perceived advantages translate into positive outcomes for consumers, and whether the perceived disadvantages of such boards can be addressed without compromising the safety and positive outcomes of the board.

## Abbreviations

ANU: Australian National University
ISG: Internet support group
NHMRC: National Health and Medical Research Council
